# Novel octapeptide containing the RGD sequence as a potential anti-SARS-CoV-2 agent: design, synthesis, and theoretical studies

**DOI:** 10.1007/s00726-025-03480-3

**Published:** 2025-11-20

**Authors:** Reiner Lemos, Orlando Ortiz, Luis Almagro, Kamil Makowski, Hortensia Rodríguez, Fernando Albericio, Margarita Suárez

**Affiliations:** 1 Laboratorio de Síntesis Orgánica, Facultad de Química, Universidad de la Habana, La Habana, 10400 Cuba; 2 Department of Surfactants and Nanobiotechnology, Institute for Advanced Chemistry of Catalonia. (IQAC-CSIC), Barcelona, 08034 Spain; 3 Centro de Investigación Biomédica en Red Bioingeniería Biomateriales y Nanomedicina (CIBER-BBN), Madrid, 28029 Spain; 4 School of Chemical Sciences and Engineering, Yachay Tech Medicinal Chemistry Research Group (MedChem-YT), Yachay Tech University, 100119 Urququi, Ecuador; 5 Peptide Science Laboratory, School of Chemistry and Physics, University of KwaZulu-Natal, Durban, 4001 South Africa; 6 Department of Organic Chemistry, University of Barcelona, Barcelona, Spain

**Keywords:** RGD motif, Integrin α_5_β_1_, DFT calculations, Solid-phase peptide synthesis

## Abstract

The design of peptide-based inhibitors targeting cell receptors represents a promising strategy in the development of antiviral agents. In this study, a novel octapeptide containing the RGD sequence was rationally designed to explore its potential interaction with integrins. The peptide was functionalized with a malonic moiety to enhance its binding capabilities and potential bioactivity. Conformational and physicochemical properties were evaluated using DFT-PBEh-3c calculations. Molecular docking studies revealed favorable interactions with the integrin α_5_β_1_, including coordination with the Mg²⁺ ion at the active site. The peptide was successfully synthesized via Fmoc-based solid-phase peptide synthesis (SPPS) and fully characterized by NMR, IR, MS, and RP-HPLC.

**Supplementary Information:**

The online version contains supplementary material available at 10.1007/s00726-025-03480-3.

## Introduction

**Fig. 1:**
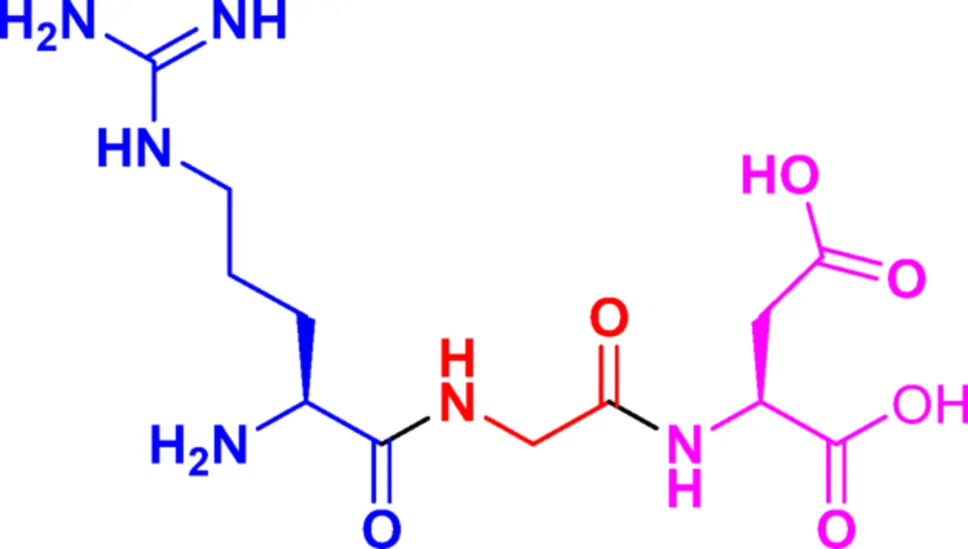
Structure of RGD sequence. Arg(R) and Asp(D) are in the L-configuration, and glycine is achiral

Arginine-glycine-aspartic (RGD) (Fig. 1) is a cell adhesion motif displayed on many extracellular matrix and plasma proteins (Colombo and Bianchi 2010). Since RGD was first identified as specific binding sites for fibronectin (FN) and the FN receptor, (Pierschbacher and Ruoslahti 1984) it has attracted widespread attention and research.

It has been reported that membrane proteins associated with extracellular matrix glycoprotein receptors on the cell surface were called integrins, which were members of the adhesion receptors (Hynes 1987). RGD plays a crucial role in cell recognition and adhesion, and has been utilized in tumor therapy and tissue engineering through recombinant and chemical methods. RGD-based ligands for integrins are studied in pathology and pharmacology (Colombo and Bianchi 2010). Furthermore, the RGD-integrin system is exploited to target cell recognition and internalization, which is applied to man-made constructs by mimicking pathogens. This system enables the study of various aspects, including diagnostics, therapeutics, and the regeneration of transplanted tissue. RGD-modified drugs and imaging agents have been investigated and developed by conjugation of the RGD peptides with a carrier device. This one has been equipped with drug molecules or reporter molecules. RGD-peptides and RGD-mimetics have also been applied to modify liposomes, polymers, and peptides by chemical means to improve the biological effects of therapeutic agents (Wang et al. 2013). Additionally, RGD-peptides were utilized in gene delivery by viral and non-viral vectors (Temming et al. 2005). The surface modification technology with fixed RGD peptides has promoted the application of integrin-mediated cell adhesion to develop tissue engineering, especially for biomaterials.

The Severe Acute Respiratory Syndrome Coronavirus 2 (SARS-CoV-2), caused by a new type of coronavirus (2019-nCoV), has emerged from China and has led to thousands of deaths in the world (Zhu et al. 2020). The viruses often bind to receptor proteins on the surface of cells to enter human cells. In the case of the SARS virus, the spike glycoprotein mediates receptor recognition and membrane fusion. The receptor binding domain (RBD), contained in the spike glycoprotein, binds directly to the peptidase domain of the angiotensin-converting enzyme 2 (ACE2)(Chen et al. 2020; Han et al. 2006).

Dakal 2021 suggests an additional mechanism of virus recognition through integrins. The pair-wise sequence alignment of SARS-CoV-2 spike protein RBD contains an RGD sequence, and SARS-CoV-2 may be attached to integrins via the RGD motif. The development of high-affinity RGD-based ligands for integrin receptors has been extensively advanced by Kessler and co-workers, whose pioneering contributions in cyclic peptide design and structure–activity relationships have set the foundation for targeting integrin α_5_β_1_ and related receptors (Kapp et al. 2016; Ludwig et al. 2021; D’Amore et al. 2023). The interaction of the RGD motif with the integrins may be involved in facilitating virus entry into host cells (Dakal 2021). The integrin α_5_β_1_ is the major cellular receptor for the extracellular matrix protein fibronectin, and it functions as a fibronectin receptor. This integrin has an RGD motif binding site located at the interface between the α_5_ and β_1_ chain, which potentially affects he binding of α_5_β_1_ with proteins or peptides (Nagae et al. 2012). Previous work has reported the antiviral properties of some peptides, with potent broad-spectrum antiviral activities (Lee et al. 2022). Interestingly, there are antiviral peptides demonstrated to exert prophylactic and therapeutic effects against coronaviruses (Mahendran et al. 2022).

Recent studies have suggested that, in addition to angiotensin-converting enzyme 2 (ACE2), certain integrins, including α_5_β_1_, may act as co-receptors that facilitate SARS-CoV-2 cell entry by recognizing the RGD motif present in the receptor-binding domain (RBD) of the spike protein (Liu et al. 2022; Cai et al. 2024). This alternative binding pathway can enhance viral adhesion to host cells, potentially increasing infectivity (Sigrist et al. 2020; Simons et al. 2021). Therefore, designing synthetic peptides that selectively bind to α_5_β_1_ could competitively block this interaction, limiting the number of integrin receptors available for spike protein engagement (Park et al. 2021). Such an approach represents a targeted strategy to disrupt a secondary viral entry mechanism and may complement therapies aimed at the ACE2–RBD interface (Simons et al. 2021; Calver et al. 2021).

Nowadays, numerous research groups have engaged in the study of therapies for this new virus, based on the understanding of the COVID-19 target-ligand interactions. Many efforts have been made to design and screen therapeutics for the current SARS-CoV-2. Therefore, this work focuses on the design and theoretical evaluation of two small peptides containing the RBD sequence, and a rational synthesis based on molecular docking results as potential anti-SARS-CoV-2 agents. To clarify the rationale behind peptide design, both synthesized peptides were engineered around the RGD motif, which is key for integrin α_5_β_1_ recognition. The surrounding amino acids, including Ala, Gly, Val, Asp, Leu, and Arg, were chosen to optimize solubility, hydrogen bonding potential, and compact conformation favorable for receptor binding. The malonic acid moiety was strategically added to promote interaction with the divalent Mg²⁺ ion at the MIDAS site of the integrin, enhancing binding affinity and specificity.

## Results and discussion

**Fig. 2:**
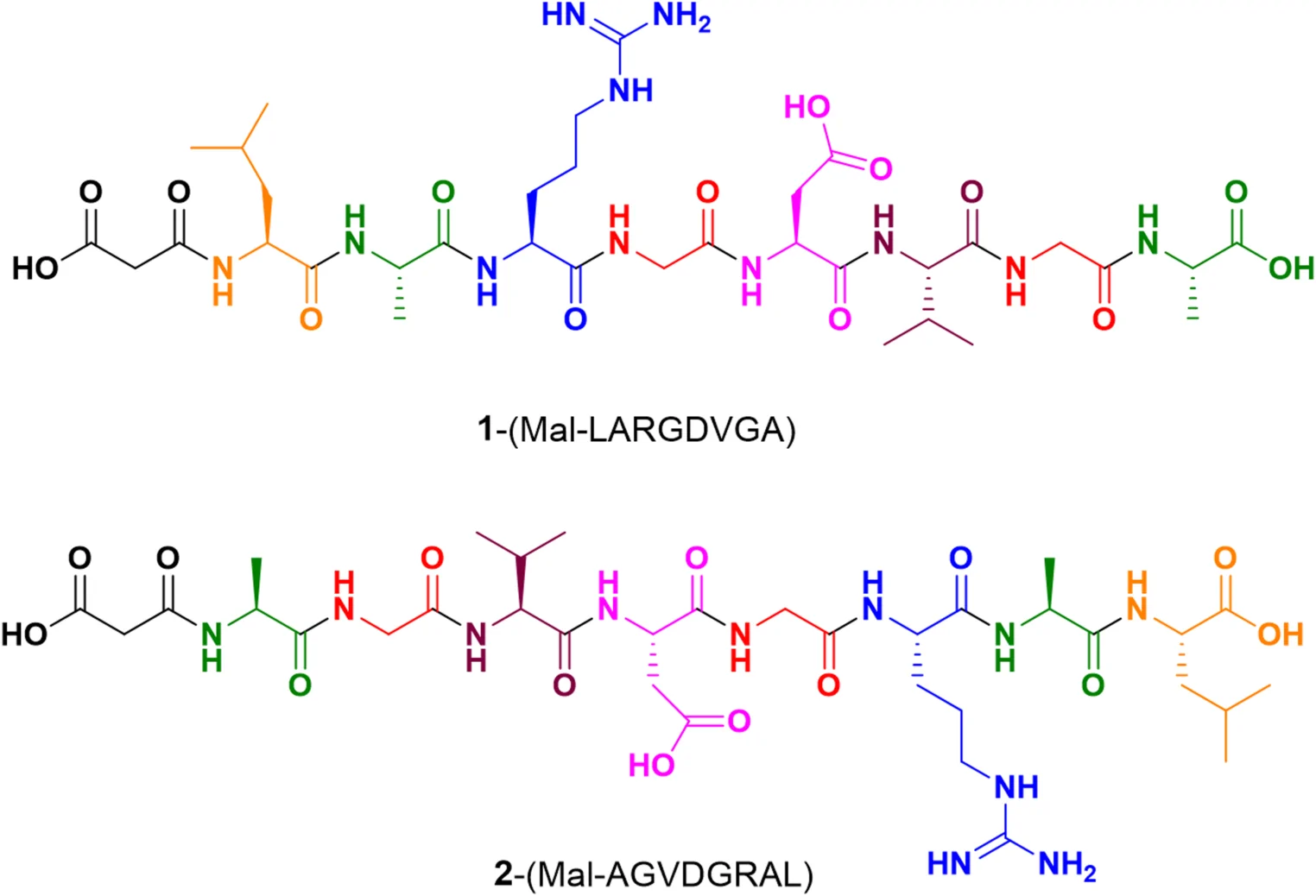
Structure of octapeptides **1** and **2** containing the RGD sequence. All amino acids are in the L-configuration, except glycine, which is achiral

In the search for new compounds as antivirals, a study was conducted on the synthesis and theoretical calculations of peptides capable of interacting with α_5_β_1_integrin receptors. Two octapeptides were designed containing the RGD sequence functionalized with a malonic moiety to evaluate their interactions with the integrin α_5_β_1_, see Fig. 2.

The presence of the malonate (Mal) moiety in a peptide would increase the number of interactions with biological receptors (Shi et al. 2024; Wilhelm et al. 2012). In addition to its ability to form stable complexes with metals and promote metal chelation of calcium and magnesium, it has potential applications in medicinal chemistry (Ojha et al. 2010). Similar strategies have been reported to enhance integrin ligand affinity (Kapp et al. 2016; Wu et al. 2019; Ludwig et al. 2021).

The sequence design also intended to explore conformational differences and interaction patterns by altering the order of residues around the RGD motif, but keeping the L-conformation of all used amino acids. The flanking residues were selected seeking to enhance solubility, conformational stability, and interaction with the integrin receptor. Specifically, the peptide includes residues such as Ala, Gly, Val, Leu, and Arg to support structural compactness and foster internal hydrogen bonding, while the malonate moiety increases the interaction potential at the MIDAS site, as was mentioned before. This method is widely used in the design of peptides to modulate recognition by biological receptors (Atzori et al. 2013).

**Fig. 3:**
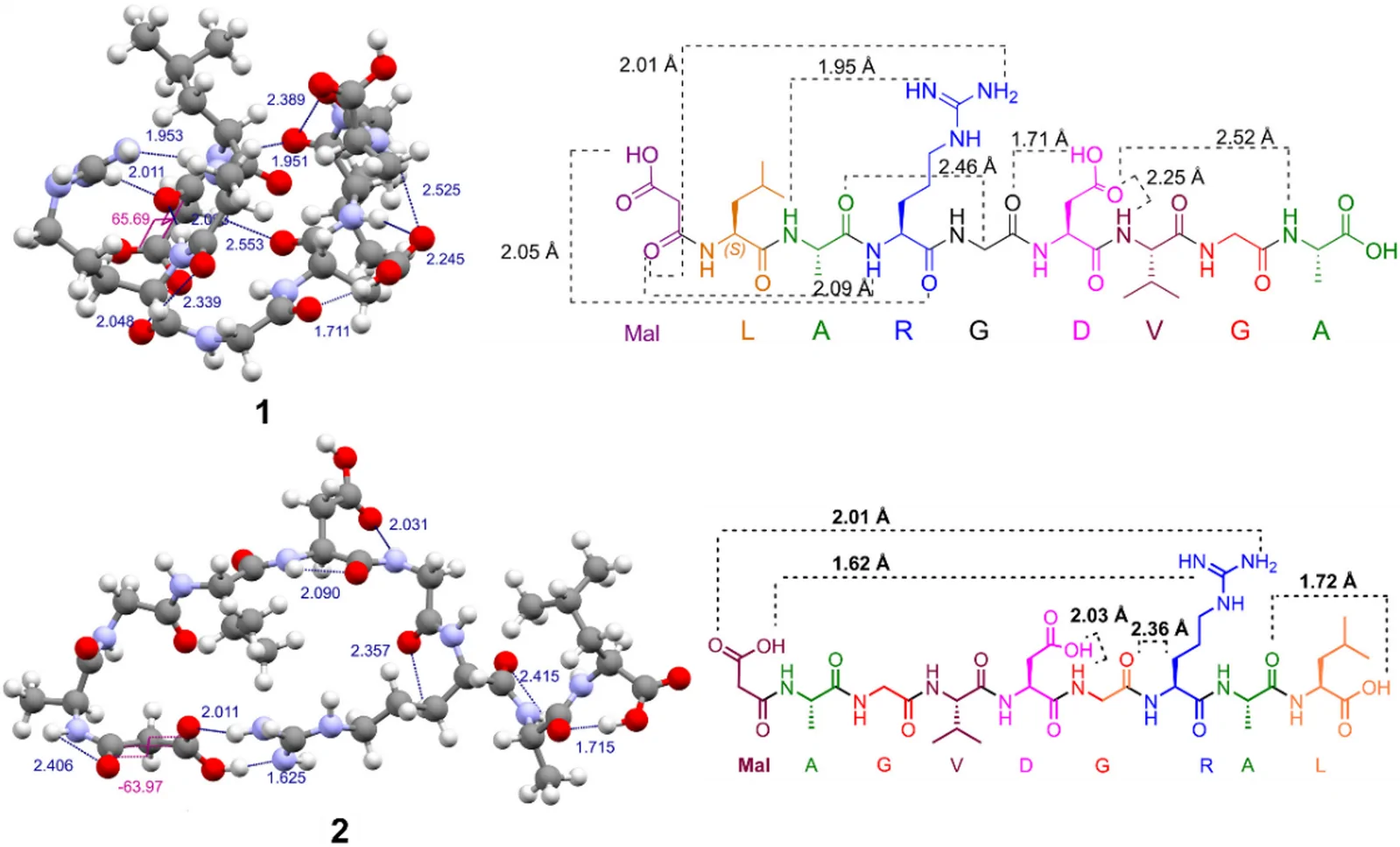
Optimized structure of compounds **1** and **2** obtained by the PBEh-3c composite scheme DFT method, distances are given in Å. 2-D representation of the most important interactions

For a better understanding of the structural properties of peptides **1** and **2**, a theoretical conformational analysis was performed. In the first place, the molecule was pre-optimized with the low-cost B97-3c (Brandenburg et al. 2018) generalized gradient approximation method. The obtained structure was further optimized using a hybrid PBEh-3c (Grimme et al. 2015) composite scheme, which presents excellent results close to the MP2 method and outperforms the popular B3LYP/6-31G approach at lower computational cost, see Fig. 3. The Cartesian atom coordinates are given in Table S1 of the Supporting Information.

Modified octapeptide **1** exhibits a highly compact structure characterized by strong interactions between residues, together with the extra interactions due to the presence of the malonic moiety. The malonic acid’s hydroxyl group is located close to arginine’s backbone carbonyl and forms a strong hydrogen bond OH···O = C, with a 2.05 Å distance. Instead, compound **2** shows a cyclic-like conformation promoted by two hydrogen bonds between the Mal fragment and the arginine residue. The carbonyl in the malonic moiety presents a synclinal relative position to each other with a torsion angle of 65.7º (**1**) and 64.0º (**2**), which permits the previously mentioned interaction with Arg residue.

Asp residue produces several strong interactions with vicinal residues such as glycine (OH···O = C, 1.71 Å) and valine and alanine residues (C = O···HN, 2.25 Å and 2.52 Å respectively) for **1**. For peptide **2**, only the aspartate residue formed hydrogen bonds with the glycine (OH···HN, 2.03 Å).

**Fig. 4:**
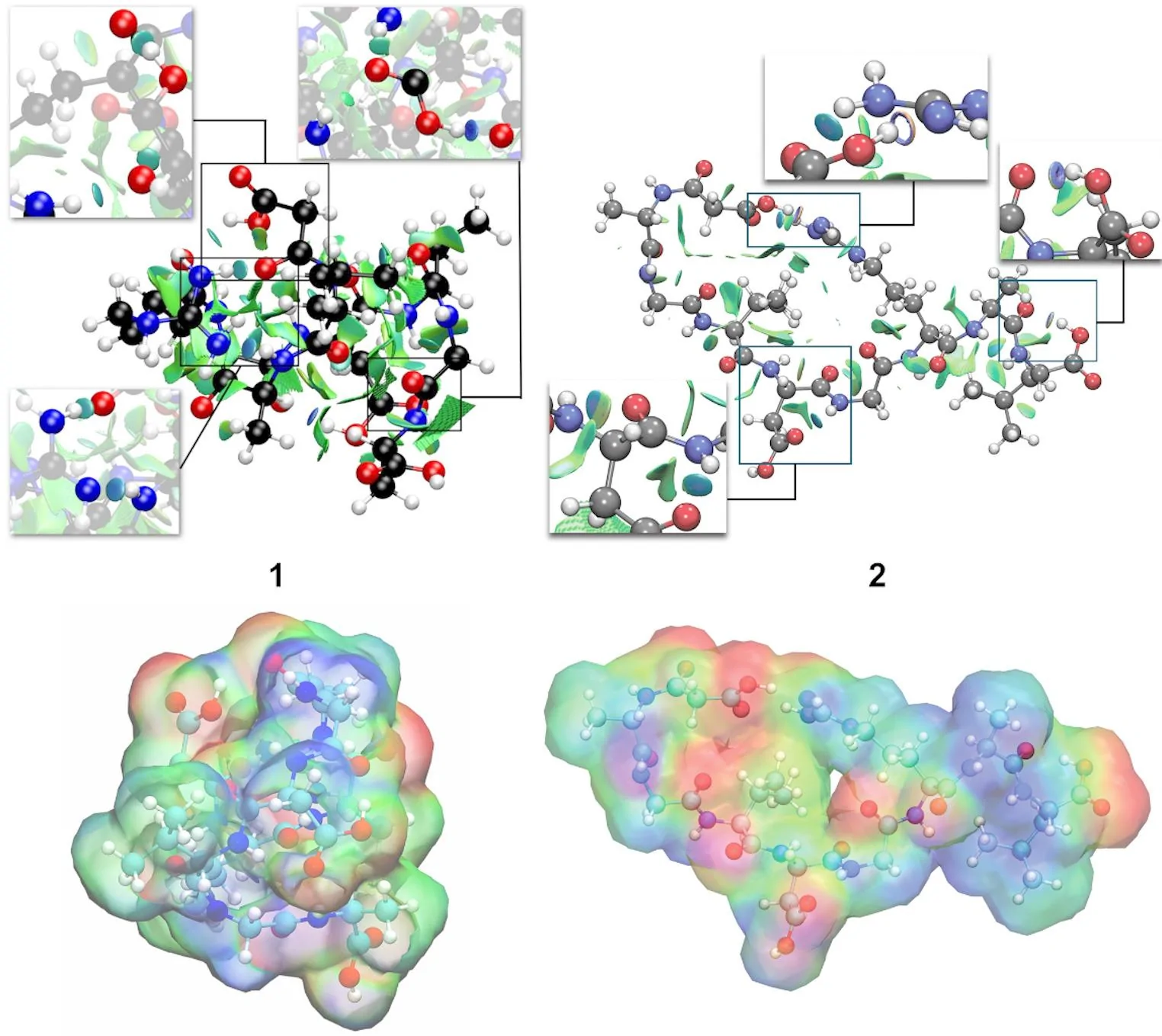
NCI representation (up) and electrostatic potential maps (down) of **1** and **2**. For better visualization, the regions with strong interactions are amplified

The analysis of non-covalent interactions (NCI) confirms the strong intramolecular interactions observed in the DFT calculations for both peptides, as shown in Fig. 4.

The green isosurfaces represent weak van der Waals interactions, and the blue ones represent strong attractive interactions. Strong intramolecular hydrogen bonds are observed between the malonate group and the guanidinium group of arginine, as well as between the free carboxyl groups and nearby amide groups, in both peptides. In Peptide **1**, the aspartate side chain forms powerful hydrogen bonds that contribute to a more compact, stable conformation compared to Peptide **2**. Moreover, the electrostatic potential maps of peptides **1** and **2** are depicted. The red zones denote sites with rich electron density, correlated mainly with carboxyl and amide groups. The positive areas (blue regions) and the green ones are found along the peptide skeletons. Therefore, the distribution of charges across the peptide suggests good water solubility.

Additional parameters were calculated to predict some physicochemical properties (see Table 1). Volume and surface area parameters confirmed the compact structure of peptide **1**, as it had lower values than peptide **2**. In the case of TPSA, this parameter is estimated mathematically from the molecule’s 2D structure, without the need to calculate actual 3D surfaces. Therefore, since both peptides have the same molecular mass and functional groups, this value coincides. Regarding solubility, hydrophilic behavior is expected from the electrostatic potential maps. The QPlogPo/w calculation suggests that both peptides exhibit hydrophilic behavior, with less pronounced behavior for peptide **1**. Overall, both peptides are expected to exhibit good bioavailability in aqueous media, which is a positive factor in future biological studies.

**Table 1:** Molecular predicted descriptors for peptides 1 and 2

Parameters (Schrödinger, Release 2021)	1	2
Volume (Å^3^)^a^	2031.93	2280.73
SASA (Å^2^) ^b^	850.65	1086.291
TPSA (Å^2^) ^c^	406.60	406.60
PSA (Å^2^)^d^	381.92	438.840
Glob ^e^	0.91	0.77
QPlogPo/w ^f^	−2.49	−2.82

^a^Total volume calculated by QikProp

^b^Solvent-accessible *surface* area calculated by QikProp

^c^Topological polar surface area calculated by the BioTriangle web server

^d^Van der Waals surface area of polar nitrogen and oxygen atoms and carbonyl carbon atoms predicted by QikProp

^e^Globularity descriptor predicted by QikProp. (Globularity is 1.0 for a spherical molecule)

^f^Predicted octanol/water partition coefficient by QikProp

A docking simulation was performed to analyze the interaction of **1** and **2** with the integrin α_5_β_1_, a target involved in the cellular recognition of SARS-CoV-2. The affinities were negative in both cases, with values of −7.2 kcal/mol (**1**) and − 7.3 kcal/mol (**2**) for the representative group of each peptide. Both peptides have a similar affinity to the receptors due to their structural similarity.

**Fig. 5:**
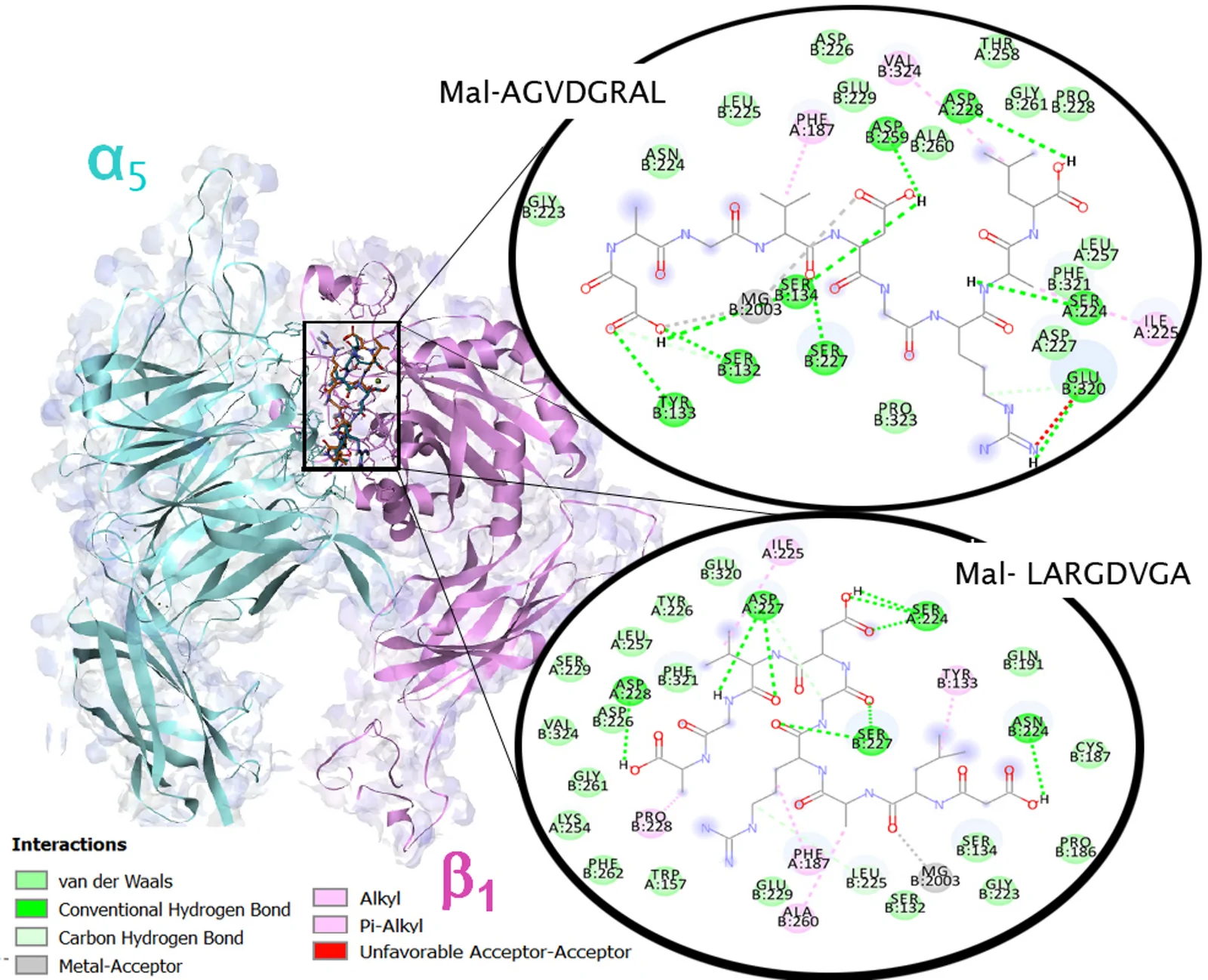
Molecular model of octapeptides **1** and **2** interactions with α_5_β_1_. 2D interacting residues are represented

Molecular docking results thus describe the general binding mode of the peptides to integrin α_5_β_1_. The hydrogen bonds and hydrophobic interactions between integrin and the ligands were visualized by employing a bidimensional interaction diagram (See Fig. 5). To validate our docking strategy, our results were compared to other docking research using known integrin α_5_β_1_ ligands derived from fibronectin-based RGD peptides (Schumacher et al. 2021). The binding poses of peptides **1** and **2** showed conserved interactions with Asp227 and coordination with the MIDAS metal center, consistent with the binding behavior observed in previous experimental and computational studies of RGD–integrin complexes (Schumacher et al. 2021; Pang et al. 2023). These results provide additional support for the predictive capability of our computational approach.

Interestingly, peptide **2** shows an H-bond with Asp227 (α5), a residue involved in the recognition of the RGD motif in the RBD protein. On the other hand, the mechanism of integrin–ligand recognition involves a direct coordinated carbonyl or carboxylate oxygen from the ligand with a divalent metal. The Mg^2+^ is coordinated by two carboxylic groups of the Mal-AGVDGRAL (**2**) peptide, while for one carbonyl group of Mal-LARGDVGA (**1**). The peptide **1** leaves a coordination site free on the metal and could be coordinated by an amino acid residue of the integrin. This ligand-Mg-protein interaction (metal ion–dependent adhesion site, MIDAS) is crucial for protein-protein inhibition as it intervenes in protein recognition. Complete Mg coordination could be more effective, but it is less selective in an inhibitor design (Gerencer and McGuffin 2023). In this type of coordination, the interaction with the metal is independent of the protein type; furthermore, this could affect other physiological functions where the metal is involved in other biological processes (Dakal 2021). Both peptides form the same number of H-bonds with the active residues.

Our results were evaluated by comparison with published crystallographic and modeling studies of fibronectin-derived RGD ligands bound to integrin α_5_β_1_. Previous work has reported conserved interactions involving the Asp residue of the RGD motif and coordination with the MIDAS metal ion, along with hydrogen bonding to residues such as Asp227 and Tyr178 (Xiong et al. 2001; Xia and Springer 2014; Schumacher et al. 2021). The docking models obtained for Peptides **1** and **2** in this study reproduce these key structural features, particularly the orientation of the RGD motif and its engagement with the MIDAS site. This agreement with experimentally determined and computationally validated integrin–ligand complexes reinforces the plausibility of our predicted binding modes.

**Scheme 1:**
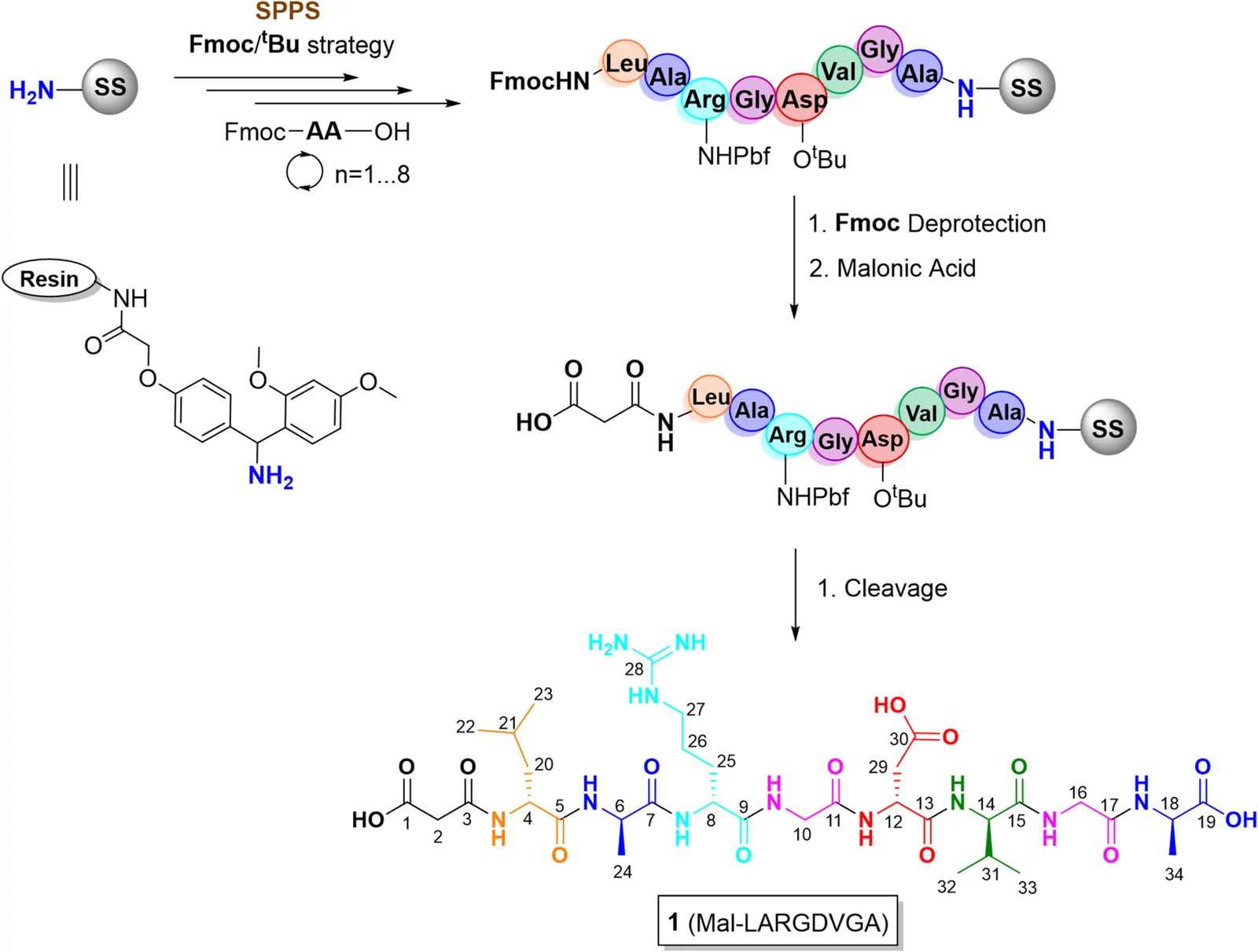
Fmoc Rink Amide MBHA resin of Mal-octapeptide **1**. Fmoc Deprotection: piperidine (20%) in DMF; Cleaved: TFA/TIS/H_2_O (95:2.5:2.5)

Theoretical studies suggest that the Mal-LARGDVGA peptide (**1**) will be a better potential inhibitor due to the number of specific interactions observed and the interaction with the magnesium atom. Taking this into account, a synthesis strategy was developed to obtain compound **1**. Octapeptide **1** was synthesized on Fmoc-Ring-Amide MBHA resin by a solid-phase procedure, using the Fmoc/tBu strategy (See Scheme 1).

The coupling of each amino acid was achieved using the activating mixture of diisopropylcarbodiimide/1-hydroxybenzotriazole (DIC/HOBt), and the completion of the reaction was verified by the Ninhydrin test. After loading the Fmoc-Ala-OH, the subsequent elongation of the peptide was achieved by stepwise coupling of the Fmoc-amino acids, such as Fmoc-Gly-OH, Fmoc-Val-OH, Fmoc-Asp(tBu)-OH, Fmoc-Gly-OH, Fmoc-Arg(Pbf)-OH, Fmoc-Ala-OH, and Fmoc-Leu-OH in this order. The deprotection of the Fmoc group was carried out using a solution of piperidine (20%) in DMF (*N*,* N*-dimethylformamide), before the next amide coupling process.

In the final stage of the reaction, a malonate fragment is added, which increases polarity and the number of strong interactions with the receptor. It also enables the generation of heterocycles and alkylated derivatives (Brosge et al. 2021) and cyclopropanations with appropriate substrates such as fullerenes (Lemos et al. 2025). The covalent binding of the malonyl group to the *N*-terminus of the peptide was realized using malonic acid and the mixture of TBTU/DIEA in DMF at room temperature. The resulting malonyl-peptide was cleaved from the resin employing a mixture of TFA/TIS/H_2_O (95:2.5:2.5), and it was precipitated with diethyl ether and centrifuged. Finally, the product was dissolved in a mixture of acetonitrile/H_2_O (1:2) and then lyophilized. Peptide **1** was obtained as a white solid powder, with an 82% yield. For details, see Experimental Section.

Structural characterization of **1** was carried out by a combination of different spectroscopic and analytical techniques, see the Experimental Section and Supporting Information. Using a combination of 1D and 2D NMR techniques enabled the signals in the ^1^H and ^13^C NMR spectra to be assigned. ^1^H-NMR spectrum shows groups of signals corresponding to the protons present in the molecule. The methylene protons of the malonate fragment appear as a singlet at δ = 3.87 ppm. Between 7.4 and 8.3 ppm are signals assignable to the protons attached to the NH protons, and in the zone 4.8–0.89 ppm, the signals corresponding to the aliphatic hydrogen are observed. Because we had achieved unambiguous assignments for the ^1^H-NMR resonances, the ^13^C-NMR resonances were assigned straightforwardly by analysis of the HSQC spectra for the protonated carbon atoms based on their chemical shift, substituent effects, and DEPT data. Quaternary carbon atoms were assigned by analysis of the HMBC spectra.

The ^13^C NMR spectrum shows the presence of the signals of three carboxyl groups at 177.8, 175.8, and 175.8 ppm, the first one assignable to the carboxyl group of the malonate fragment. The other eight signals between 174.7 and 170.6 ppm are assigned to the C = O of the amide groups present. The carbon of the C = NH group appears at 158.7 ppm, and the signal of the carbon of the methylene group of the malonate fragment is located at 43.8 ppm. The other 21 signals appearing in the spectrum were unambiguously assigned to the remaining carbon atoms in the molecule. In the ^15^N-^1^H correlation HMBC spectrum, the signals corresponding to the nitrogen atoms present in the molecule can be observed at 127.21, 121.65, 119.69, 116.97, 116.71, 115.82, 108.31, 105.54, 101.22, 101.13 ppm (see Figure S7), verifying the assignment of the proton bonded to the nitrogen atoms.

The malonyl-octapeptide **1** was analyzed through reverse phase-high performance liquid chromatography (RP-HPLC), determining that the peptide has high purity (See Figure S9) and was characterized by electrospray ionization time of flight mass spectrometry (TOF-MS ESI), which supported the proposed structure. The ESI spectrum shows a peak at *m/z* = 843.51, which corresponds to its molecular weight, see Figure S10 in the Supporting Information. The IR spectrum shows the broad feature the 3425 cm^−1^ characteristic of the O–H stretching band of the acid, which is known to be in dimeric form due to hydrogen bonding. The CH_2_ and CH_3_ symmetric and asymmetric stretching vibrations are detected from 2960 to 2855 cm^−1^, the carbonyl bands appear at 1740 cm^−1^, see Figure S8 in the Supporting Information.

Also, the thermal stability of octapeptide **1** was evaluated by thermogravimetric analysis (Figure S11, Supporting Information). The thermograms exhibit three main weight losses. The first one, between 100 and 180 °C, is presumably due to the loss of solvents like water and the possible decarboxylation of the malonic fragment. (Caires et al. 2010) The range from 180 to 250 °C could be considered the remainder of the decomposition of terminal carboxyl groups and the elimination of ammonia from amine residues. From 250 °C onwards, a continuous loss related to the breaking of peptide bonds is observed, which is a common behavior of small peptides. In summary, the peptide can be considered stable for biomedical applications, as it is stable up to 100 °C, a temperature at which it begins to decompose depending on its structure.

The use of in silico calculations in the design of a novel octapeptide containing an RGD sequence and potential inhibitory activity against SARS-CoV-2 has proven to be an efficient and cost-effective strategy. This computational approach allowed us to focus subsequent efforts on the most promising structures, peptide **1**, based on favorable binding affinities and interaction profiles. Nevertheless, while these in silico results provide a potential activity of the designed peptide, further experimental validation through in vitro and in vivo tests will be necessary to validate its biological efficacy and potential therapeutic relevance.

### Experimental section

General Methods. Solvents were dried by standard procedures. All reagents were of commercial quality and were used as supplied unless otherwise specified. The analysis of the purity of the malonyl-peptide synthesized was carried out by RP-HPLC in a Shimadzu equipment I-760-287, column C18 (Vydac, 4.6 × 150 mm, 5 μm), and the injection volume of 100 µL. The mobile phases used were 0.1 (v/v) of TFA in water (A) and 0.05% (v/v) of TFA in acetonitrile (B). A linear gradient of 5 to 60% of B during 35 min., with a flux of 0.8 mL/min, was used to elute the analyte. The retention time (R_t_) and the peak area (Pas) were determined at a wavelength of 226 nm. FTIR spectra were carried out using ATR of the solid compounds. The ESI-MS spectra were obtained in orthogonal hybrid configuration spectrometers Q-Tof 1 or Q-Tof 2 (Micromass, England) with a nanospray ionization source. The voltages of the borosilicate capillary and the inlet cone were set at 900 and 35 V, respectively. A solution of sodium and cesium iodide was used as a reference for the calibration of the spectrometer. The Masslynx version 4.1 program (Micromass, England) was used for the processing of mass spectra. The accepted error for the determination of the experimental MM was 0.01% of the theoretical MM. ^1^H NMR spectra were recorded in methanol-*d*₄ (CD₃OD) at 700 MHz, and ^13^C NMR at 175 MHz with a Bruker Avance 700 instrument. The one-bond heteronuclear correlation (HSQC), the long-range ^1^-^13^ C correlation (HMBC), and ^1^-^15^ N correlation (HSQC) spectra were obtained by use of the inv4gs and the inv4gslplrnd programs with the Bruker software. TGA analyses were carried out under air and nitrogen in a TATGA-Q500 apparatus. The sample (∼ 0.5 mg) was introduced inside a platinum crucible and equilibrated at 90 °C, followed by a 10 °C/min ramp between 90 and 1000 °C.

### Computational methods

All molecules were built with Avogadro (Hanwell et al. 2012), and DFT calculations were performed with ORCA 4.2.1 (Neese et al. 2020). All structures were pre-calculated using the B97-3c(Brandenburg et al. 2018) composite scheme as a fast DFT approach. Then structures were optimized using DFT with a hybrid functional PBEh-3c composite scheme, which includes a polarized double-ζ basis set and London dispersion correction with Becke–Johnson damping (D3BJ) method (Grimme et al. 2015). This method presents very good accuracy at a reasonable computational cost. Very tight convergence as keywords were employed, improving the numerical precision as Orca implantation permits. The Lebedev302 grid (Grid4) during the SCF iterations and Lebedev434 (Grid 5) as a final grid for the final energy evaluation after SCF convergence were set for integration precision. No imaginary frequencies were found after calculations of frequencies on optimized structures using the same level of theory. Optimized structures were used for the calculation of properties and visualization of the electrostatic potential. Most properties were predicted by QikProp from Schrödinger (Schrödinger Release 2024-4: Maestro, Schrödinger 2024) packaged using DFT optimized structures. For the molecular electrostatic map, first the mep.py Python script written by Marius Retegan (Retegan 2019; Mep.Py. mep.py (source code), github repository, https://gist.github.com/mretegan/5501553. n.d.) was used for cube file preparation, and then visualized using VMD 1.9.3 (Humphrey et al. 1996) Non-covalent interactions were generated by Multiwfn 3.7.(Lu and Chen 2012) program, and then NCIPLOT 4.0 (Boto et al. 2020) software was used. The VMD program was used together with vmd script generated by NCIPLOT, the visualization of NCI.

### Molecular modeling

The PDB file of the integrin α_5_β_1_ (PDB code: 3VI3) was retrieved from the Protein Data Bank (http://rcsb.org). Ligand-optimized structures were obtained from the DFT method. The PDB files of proteins and ligands were converted to PDBQT format with AutoDockTools. The simulation box with size 40 × 50 × 72 Å^3^ was positioned around the active site, matching the center of the ligands with the center of the simulation box. Docking simulations were performed using AutoDock 4.2. (Morris et al. 2009) Figures graphically representing the mode of interaction were prepared using Discovery Studio Visualizer 2020.

## Synthesis and characterization

### Solid phase peptide synthesis of the malonyl-octapeptide (1)

#### General resin preparation

Fmoc Rink amide MBHA resin (0.015 mmol) was placed in a peptide synthesis vessel, swollen in DMF, and deprotected with 5 ml of 20% piperidine/DMF for 4 min. Washings between the first deprotection, coupling, and subsequent deprotection steps were carried out with DMF (5 × 0.5 min) and DCM (5 × 0.5 min) using 10 ml of solvent/g of resin each time.

The peptide was synthesized manually on MBHA^®^ resin, functionalized with the spacer Am (4-(2,4-methoxy benzhydryl)phenol acetic acid), by a stepwise solid-phase procedure (Fmoc-Am-MBHA, 0.50 g, 0.48 mmol/g, 0.35 mmol) using the Fmoc/tBu (fluoren-9-ylmethyloxycarbonyl/tert-buthyl) strategy. The coupling of each amino acid was achieved using the activating mixture of DIC (218 µL, 1.38 mmol)/HOBt (diisopropylcarbodiimide/1-hydroxybenzotriazole). The completion of the reaction was verified by the ninhydrin test. All amino acids are L-conformation, except Gly, which is achiral. The order of addition of each amino acid was: Fmoc-Ala-OH x H_2_O (0.46 g, 1.40 mmol), Fmoc-Gly-OH (0.42 g, 1.41 mmol), Fmoc-Val-OH (0.48 g, 1.41 mmol), Fmoc-Asp(tBu)-OH (0.58 g, 1.41 mmol), Fmoc-Gly-OH (0.42 g, 1.41 mmol), Fmoc-Arg(Pbf)-OH (0.91 g, 1.40 mmol), Fmoc-Ala-OH x H_2_O (0.46 g, 1.40 mmol), Fmoc-Leu-OH (0.49 g, 1.40 mmol). The successive deprotection of the Fmoc group was carried out using a solution of piperidine (20%) in DMF (*N*,* N-dimethylformamide*) for 20 min. Afterward, the peptide was purified in order to get rid of the excess of piperidine, through four washes of 1 min for each one with DMF. The covalent binding of the malonyl group to the *N*-terminus of the peptide was carried out using malonic acid (0.15 g, 1.44 mmol) and the mixture of *O*-(Benzotriazol-1-yl)-*N*,* N*,*N´N´*-tetramethyluronium tetrafluoroborate, TBTU (0.45 g, mmol/g), and *N*,* N*-Diisopropylethylamine, DIEA (356 µL) in DMF at room temperature. The resulting malonyl-peptide was cleaved from the resin using the mixture trifluoroacetic acid/triisopropylsilane/water (TFA/TIS/H2O, 95:2.5:2.5) for 2 h. It was then precipitated over diethyl ether at −80 °C and centrifuged. Finally, the product was dissolved in a mixture of acetonitrile/H_2_O (1:2) and then lyophilized. The product was isolated as a white solid. Yield: 82% (330 mg, 0.82 mmol).

**Figure:**
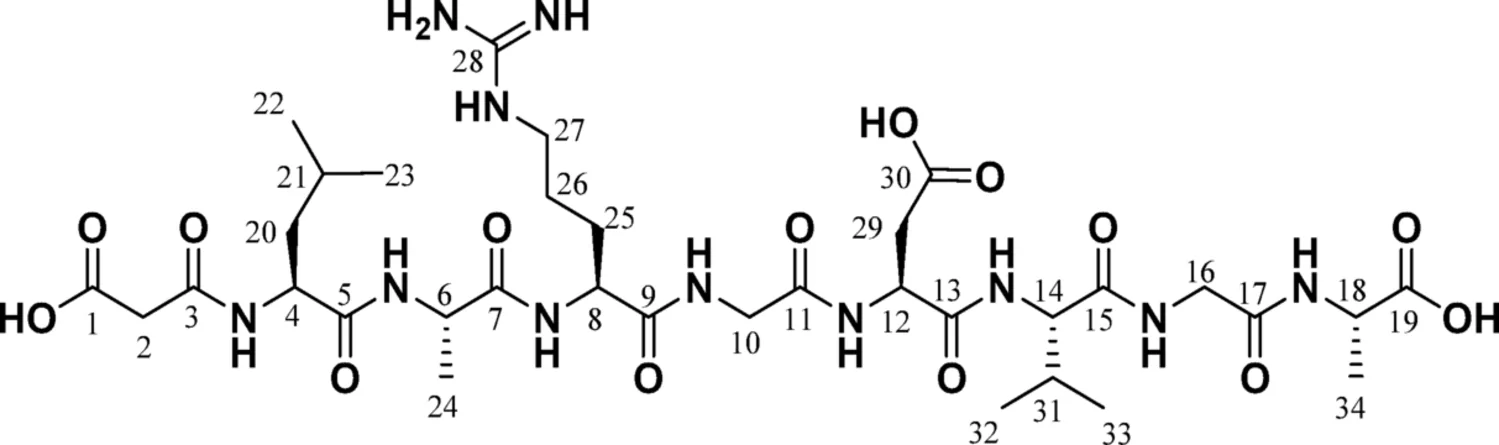

ATR-FTIR: *ν* 2423, 2959, 2925, 2855, 1740 (C = O), 1462, 1492, 1462, 1186, 1061, 969 cm^−1^. ^1^H NMR (700 MHz, CD_3_OD) δ 8.74 (d, *J* = 5.9 Hz, 1H, NH), 8.28 (m, 2 H, HA-NH_2,_ NH), 8.26 (d, *J* = 5.6 Hz, 1H, NH), 8.22 (d, *J* = 7.4 Hz, 1H, NH), 8.18 (t, *J* = 5.8 Hz, 1H, NH), 7.97 (d, *J* = 7.2 Hz, 2 H, HB-NH_2_, NH), 7.85 (d, *J* = 6.9 Hz, 1H, NH), 7.46 (s, 1H, NH), 7.40 (t, *J* = 5.3 Hz, 1H, NH), 7.08 (s, 1H, NH), 4.75 (q, *J* = 7.1 Hz, 1H, H12), 4.32 (m, 1H, H18), 4.29–4.24 (m, 3 H, H4, H6, H8), 4.08 (m, 1H, H14), 3.90–3.81 (m, 4 H, H2, H16), 3.48 (d, *J* = 16.2 Hz, 1H, H10a), 3.32 (d, *J* = 16.0 Hz, 1H, H10b), 3.20 (q, *J* = 6.7 Hz, 2 H, H27), 2.92 (dd, *J* = 17.0, 6.5 Hz, 1H, H29a), 2.78 (dd, *J* = 17.0, 7.1 Hz, 1H, H29b), 2.20 (m, 1H, H31), 1.91 (m, 1H, H25a), 1.81–1.73 (m, 2 H, H21, H25b), 1.70–1.57 (m, 4 H, H26, H20), 1.41 (d, *J* = 7.2 Hz, 3 H, H24), 1.38 (d, *J* = 7.4 Hz, 3 H, H34), 0.99 (m, 9 H, H23, H32, H33), 0.93 (d, *J* = 6.6 Hz, 3 H, H22).

^13^C{1 H} NMR (176 MHz, CD_3_OD) δ 177.9 (COOH), 175.8 (COOH), 175.8 (COOH), 174.8 (C = O), 174.5 (C = O), 174.1 (C = O), 173.6 (C = O), 172.8 (C = O), 171.9 (C = O), 171.4 (C = O), 170.6 (C = O), 158.7 (C28), 61.4 (C14), 55.0 (C4), 54.9 (C8), 51.6 (C12), 51.5 (C6), 50.4 (C18), 43.9 (C16), 43.8 (C2), 43.1 (C10), 42.0 (C27), 41.0 (C20), 36.3 (C29), 31.0 (C31), 29.3 (C25), 26.1 (C26), 25.8 (C21), 23.3 (C23), 21.6 (C22), 19.6 (C32), 18.72 (C33), 18.0 (C34), 17.00 (C24). ^15^N{1 H} NMR (71 MHz, CD_3_OD) δ 127.21, 121.65, 119.69, 116.97, 116.71, 115.82, 108.31, 105.54, 101.22, 101.13. MS ESI-TOF: *m/z*: M^+^ Calcd for C_34_H_57_N_11_O_14_: 843.48; Found: 843.51. Anal. Calcd for C_34_H_57_N_11_O_14_: C, 48.39; H, 6.81; N, 18.26; Found: C, 48.42; H, 6.83; N, 18.30.

## Conclusions

Two octapeptides containing the RGD sequence and a malonate moiety were designed and theoretically evaluated as potential inhibitors of the SARS-CoV-2 infection. Computational analysis demonstrated that peptide **1** exhibits favorable intramolecular interactions and enhanced binding to integrin α_5_β_1_ through coordination with the Mg²⁺ ion at the MIDAS site. Peptide **1** was successfully synthesized by solid-phase peptide synthesis and fully characterized, confirming its chemical structure and purity. While in silico studies suggest that peptide **1** may serve as a potential integrin-targeting antiviral agent, further in vitro and in vivo studies are needed to confirm its therapeutic potential.

## Supplementary Information

Below is the link to the electronic supplementary material.

Supplementary Material 1

## Data Availability

All data are included within this paper and its Supplementary Information files. Further information can be requested to the authors.

## References

[CR1] Atzori A, Baker AE, Chiu M, Bryce RA, Bonnet P (2013) Effect of sequence and stereochemistry reversal on p53 peptide mimicry. PLoS ONE 8:e68723. 10.1371/journal.pone.0068723 23922660 10.1371/journal.pone.0068723 PMC3726663

[CR2] Boto R, Peccati F, Laplaza R, Quan C, Carbone A, Piquemal JP, Maday Y, Contreras-García J (2020) NCIplot4: a new step towards a fast quantification of noncovalent interactions. ChemRxiv. https://chemrxiv.org/engage/chemrxiv/article-details/60c747ce9abda22e30f8c9a4 10.1021/acs.jctc.0c00063 32470306

[CR3] Brandenburg JG, Bannwarth C, Hansen A, Grimme S (2018) B97-3c: a revised low-cost variant of the B97-D density functional method. J Chem Phys 148:064104. 10.1063/1.5012601 29448802 10.1063/1.5012601

[CR4] Brosge F, Singh P, Almqvist F, Bolm C (2021) Selected applications of meldrum’s acid - a tutorial. Org Biomol Chem 19:5014–5027. 10.1039/D1OB00395J 34019615 10.1039/d1ob00395j

[CR5] Cai Z, Bai H, Ren D et al (2024) Integrin αvβ1 facilitates ACE2-mediated entry of SARS-CoV-2. Virus Res 339:199251. 10.1016/j.virusres.2023.199251 37884208 10.1016/j.virusres.2023.199251 PMC10651773

[CR6] Caires FJ, Lima LS, Carvalho CT, Giagio RJ, Ionashiro M (2010) Thermal behaviour of malonic acid, sodium malonate and its compounds with some bivalent transition metal ions. Thermochim Acta 497:35–40. 10.1016/j.tca.2009.08.013

[CR7] Calver J, Joseph C, John A et al (2021) S31 The novel coronavirus SARS-CoV-2 binds RGD integrins and upregulates avb3 integrins in Covid-19 infected lungs. Thorax 76:A22–A23. 10.1136/thorax-2020-btsabstracts.37

[CR8] Chen Y, Liu Q, Guo D (2020) Emerging coronaviruses: genome structure, replication, and pathogenesis. J Med Virol 92:418–423. 10.1002/jmv.25681 31967327 10.1002/jmv.25681 PMC7167049

[CR9] Colombo M, Bianchi A (2010) Click chemistry for the synthesis of RGD-containing integrin ligands. Molecules 15:178–197. 10.3390/molecules15010178 20110882 10.3390/molecules15010178 PMC6256992

[CR10] D’Amore VM, Donati G, Lenci E et al (2023) Molecular view on the Irgd peptide binding mechanism: implications for integrin activity and selectivity profiles. J Chem Inf Model 63:6302–6315 37788340 10.1021/acs.jcim.3c01071 PMC10598797

[CR11] Dakal TC (2021) SARS-CoV-2 attachment to host cells is possibly mediated via RGD-integrin interaction in a calcium-dependent manner and suggests pulmonary EDTA chelation therapy as a novel treatment for COVID-19. Immunobiology 226:152021. 10.1016/j.imbio.2020.152021 33232865 10.1016/j.imbio.2020.152021 PMC7642744

[CR12] Gerencer M, McGuffin LJ (2023) Are the integrin binding motifs within SARS CoV-2 spike protein and MHC class II alleles playing the key role in COVID-19? Front Immunol 14:1177691. 10.3389/fimmu.2023.1177691 37492575 10.3389/fimmu.2023.1177691 PMC10364474

[CR13] Grimme S, Brandenburg JG, Bannwarth C, Hansen A (2015) Consistent structures and interactions by density functional theory with small atomic orbital basis sets. J Chem Phys 143:054107. 10.1063/1.4927476 26254642 10.1063/1.4927476

[CR14] Han DP, Penn-Nicholson A, Cho MW (2006) Identification of critical determinants on ACE2 for SARS-CoV entry and development of a potent entry inhibitor. Virology 350:15–25. 10.1016/j.virol.2006.01.029 16510163 10.1016/j.virol.2006.01.029 PMC7111894

[CR15] Hanwell MD, Curtis DE, Lonie DC, Vandermeersch T, Zurek E, Hutchison GR (2012) Avogadro: an advanced semantic chemical editor, visualization, and analysis platform. J Cheminform 4:17. 10.1186/1758-2946-4-17 22889332 10.1186/1758-2946-4-17 PMC3542060

[CR16] Humphrey W, Dalke A, Schulten K (1996) VMD: visual molecular dynamics. J Mol Graph 14:33–38. 10.1016/0263-7855(96)00018-5 8744570 10.1016/0263-7855(96)00018-5

[CR17] Hynes RO (1987) Integrins: a family of cell surface receptors. Cell 48:549–554. 10.1016/0092-8674(87)90233-9 3028640 10.1016/0092-8674(87)90233-9

[CR18] Kapp TG, Rechenmacher F, Neubauer S et al (2016) A comprehensive evaluation of the activity and selectivity profile of ligands for RGD-binding integrins. Sci Rep 6:39805. 10.1038/srep39805 10.1038/srep39805 PMC5225454 28074920

[CR19] Lee YC, Shirkey JD, Park J, Bisht K, Cowan AJ (2022) An overview of antiviral peptides and rational biodesign considerations. BioDesign Res 2022:9898241. 10.34133/2022/9898241 10.34133/2022/9898241 PMC10521750 37850133

[CR20] Lemos R, Pérez-Badell Y, De Nisco M, Carpentieri A, Suárez M, Pedatella S (2025) Organic chimeras based on selenosugars, steroids, and fullerenes as potential inhibitors of the β-amyloid peptide aggregation. ChemPlusChem 90:e202400404. 10.1002/cplu.202400404 39235155 10.1002/cplu.202400404

[CR21] Liu J, Lu F, Chen Y, Plow E, Qin J (2022) Integrin mediates cell entry of the SARS-CoV-2 virus independent of cellular receptor ACE2. J Biol Chem 298:101710. 10.1016/j.jbc.2022.101710 35150743 10.1016/j.jbc.2022.101710 PMC8828381

[CR22] Lu T, Chen F (2012) Multiwfn: a multifunctional wavefunction analyzer. J Comput Chem 33:580–592. 10.1002/jcc.22885 22162017 10.1002/jcc.22885

[CR23] Ludwig BS, Kessler H, Kossatz S, Reuning U (2021) RGD-binding integrins revisited: how recently discovered functions and novel synthetic ligands (re-)shape an ever-evolving field. Cancers 13:1711–1756. 10.3390/cancers13071711 33916607 10.3390/cancers13071711 PMC8038522

[CR24] Mahendran SAK, Lim YS, Fang CM, Loh HS, Le CF (2022) The potential of antiviral peptides as COVID-19 therapeutics. Front Pharmacol 11:575444. 10.3389/fphar.2020.575444 10.3389/fphar.2020.575444 PMC7522797 33041819

[CR25] Morris GM, Huey R, Lindstrom W, Sanner MF, Belew RK, Goodsell DS, Olson AJ (2009) Software news and updates AutoDock4 and AutoDockTools4: automated docking with selective receptor flexibility. J Comput Chem 30:2785–2791. 10.1002/jcc.21256 19399780 10.1002/jcc.21256 PMC2760638

[CR26] Nagae M, Re S, Mihara E, Nogi T, Sugita Y, Takagi J (2012) Crystal structure of α5β1 integrin ectodomain: atomic details of the fibronectin receptor. J Cell Biol 197:131–140. 10.1083/jcb.201111077 22451694 10.1083/jcb.201111077 PMC3317794

[CR27] Neese F, Wennmohs F, Becker U, Riplinger C (2020) The ORCA quantum chemistry program package. J Chem Phys 152(22):224108. 10.1063/5.0004608 32534543 10.1063/5.0004608

[CR28] Ojha B, Singh AK, Adhikari MD, Ramesh A, Das G (2010) 2-alkylmalonic acid: amphiphilic chelator and a potent inhibitor of metalloenzyme. J Phys Chem A. 10.1021/jp1013847 10.1021/jp1013847 20669918

[CR29] Pang X, He X, Qiu Z, Zhang H, Xie R, Liu Z, Gu Y, Zhao N, Xiang Q, Cui Y (2023) Targeting integrin pathways: mechanisms and advances in therapy. Sig Transduct Target Ther 8:1–42. 10.1038/s41392-022-01259-6 10.1038/s41392-022-01259-6 PMC9805914 36588107

[CR30] Park EJ, Myint PK, Appiah MG et al (2021) The spike glycoprotein of sars-cov-2 binds to β1 integrins expressed on the surface of lung epithelial cells. Viruses 13:645–656. 10.3390/V13040645 33918599 10.3390/v13040645 PMC8069079

[CR31] Pierschbacher MD, Ruoslahti E (1984) Cell attachment activity of fibronectin can be duplicated by small synthetic fragments of the molecule. Nature 309:30–33. 10.1038/309030a0 6325925 10.1038/309030a0

[CR32] Retegan M (2019) Mep.Py. mep.py (source code), GitHub repository. https://gist.github.com/mretegan/5501553. Accessed 3 Dec 2024

[CR33] Schrödinger (2021) Release 2023-2: QikProp. Schrödinger LLC: New York

[CR34] Schrödinger (2024) Release 2024-4: maestro. Schrödinger LLC: New York

[CR35] Schumacher S, Takagi J, Biertümpfel C, Mizuno N (2021) Structural insights into integrin α5β1 opening by fibronectin ligand. Sci Adv 7:eabe9716. 10.1126/sciadv.abe9716 33962943 10.1126/sciadv.abe9716 PMC8104898

[CR36] Shi C, Dai J, Chang L, Xu W, Huang C, Zhao Z, Li H, Zhu L, Xu Y (2024) Design, synthesis and structure-activity relationship of malonic acid non-nucleoside derivatives as potent CD73 inhibitors. Bioorg Med Chem Lett 112:129946. 10.1016/j.bmcl.2024.129946 39226996 10.1016/j.bmcl.2024.129946

[CR37] Sigrist CJ, Bridge A, Le Mercier P (2020) A potential role for integrins in host cell entry by SARS-CoV-2. Antivir Res 177:104759–104761. 10.1016/j.antiviral.2020.104759 32130973 10.1016/j.antiviral.2020.104759 PMC7114098

[CR38] Simons P, Rinaldi DA, Bondu V et al (2021) Integrin activation is an essential component of SARS-CoV-2 Integrin activation is an essential component of SARS-CoV-2 infection infection. Sci Rep 11:20398. 10.1038/s41598-021-99893-7 34650161 10.1038/s41598-021-99893-7 PMC8516859

[CR39] Temming K, Schiffelers RM, Molema G, Kok RJ (2005) RGD-based strategies for selective delivery of therapeutics and imaging agents to the tumour vasculature. Drug Resist Updat 8:381–402. 10.1016/j.drup.2005.10.002 16309948 10.1016/j.drup.2005.10.002

[CR40] Wang F, Li Y, Shen Y, Wang A, Wang S, Xie T (2013) The functions and applications of RGD in tumor therapy and tissue engineering. Int J Mol Sci 14:13447–13462. 10.3390/ijms140713447 23807504 10.3390/ijms140713447 PMC3742196

[CR41] Wilhelm A, Lopez-Garcia LA, Busschots K, Frö W, Maurer F, Boettcher S, Zhang H, Jörg O, Schulze O, Biondi RM, Engel M (2012) 2-(3-oxo-1,3-diphenylpropyl)malonic acids as potent allosteric ligands of the PIF pocket of phosphoinositide-dependent kinase-1: development and prodrug concept. J Med Chem 55:9817–9830. 10.1021/jm3010477 23106316 10.1021/jm3010477

[CR42] Wu PH, Opadele AE, Onodera Y, Nam JM (2019) Targeting integrins in cancer nanomedicine: applications in cancer diagnosis and therapy. Cancers (Basel) 11:1783–1807. 10.3390/cancers11111783 31766201 10.3390/cancers11111783 PMC6895796

[CR43] Xia W, Springer TA (2014) Metal ion and ligand binding of integrin α_5_β_1_. Proc Natl Acad Sci U S A 111:17863–17868. 10.1073/pnas.1420645111/suppl_file/pnas.1420645111.sm01.mpg 25475857 10.1073/pnas.1420645111 PMC4273411

[CR44] Xiong JP, Stehle T, Diefenbach B et al (2001) Crystal structure of the extracellular segment of integrin α_V_β_3_. Science 294:339–345. 10.1126/science.1064535 11546839 10.1126/science.1064535 PMC2885948

[CR45] Zhu N, Zhang D, Wang W, Li X, Yang B, Song J, Zhao X, Huang B, Shi W, Lu R, Niu P, Zhan F, Ma X, Wang D, Xu D, Wu D, Gao GF, Tan W (2020) A novel coronavirus from patients with pneumonia in China. N Engl J Med 382:727–733. 10.1056/nejmoa2001017 31978945 10.1056/NEJMoa2001017 PMC7092803

