# Supplementary material for: Novel octapeptide containing the RGD sequence as a potential anti-SARS-CoV-2 agent: design, synthesis, and theoretical studies
Source: Amino Acids. 2025 Nov 20;57:56. doi: 10.1007/s00726-025-03480-3 (PMC12657584; doi:10.1007/s00726-025-03480-3)
Supplement: Supplementary file 1 [file 726_2025_3480_MOESM1_ESM.docx]

Supporting Information

Novel Octapeptide Containing the RGD Sequence as a Potential Anti-SARS-CoV-2 Agent: Design, Synthesis, and Theoretical Studies

Reiner Lemos,^[1]*^ Orlando Ortiz,^[1]^ Luis Almagro,^[1]^ Kamil Makowski,^[2]^ Hortensia Rodríguez,^[3]^ Fernando Albericio,^[4]*^ Margarita Suarez^[1]*^

^[1]^ Laboratorio de Síntesis Orgánica, Facultad de Química, Universidad de la Habana, 10400-La Habana, Cuba.

^[2]^ Department of Surfactants and Nanobiotechnology, Institute for Advanced Chemistry of Catalonia. (IQAC-CSIC), 08034 Barcelona (Spain) and Centro de Investigación Biomédica en Red Bioingeniería Biomateriales y Nanomedicina (CIBER-BBN) 28029-Madrid (Spain)

^[3]^ Yachay Tech Medicinal Chemistry Research Group (MedChem-YT), School of Chemical Sciences and Engineering, Yachay Tech University, 100119-Urququi, Ecuador.

^[4]^School of Chemistry and Physics, University of KwaZulu-Natal, Durban 4001, South Africa and Department of Organic Chemistry, University of Barcelona, Barcelona, Spain.

Corresponding authors: [reinier.lemos@fq.uh.cu](mailto:reinier.lemos@fq.uh.cu) (RL), [albericio@ub.edu](mailto:albericio@ub.edu); [albericio@ukzn.ac.za](mailto:albericio@ukzn.ac.za) (FA), [msuarez@fq.uh.cu](mailto:msuarez@fq.uh.cu) (MS)

[**1.**	**Spectroscopy characterization**	S1](#_Toc201834149)

[1.1.	Spectra of peptide **1** (Mal-LARGDVGA)	S1](#_Toc201834150)

[**Figure S1.** ^1^H NMR spectrum of peptide Mal-LARGDVGA.	S1](#_Toc201834151)

[**Figure S2.** ^13^C NMR spectrum of peptide **1**.	S2](#_Toc201834152)

[**Figure S4.** HSQC spectrum of peptide **1**	S3](#_Toc201834153)

[**Figure S5.** HMBC spectrum of peptide **1**	S3](#_Toc201834154)

[**Figure S6.** COSY spectrum of peptide **1**.	S4](#_Toc201834155)

[**Figure S7.** ^15^N-^1^H correlation HMBC spectrum of peptide **1**	S4](#_Toc201834156)

[**Figure S8.** FTIR spectrum of peptide **1**.	S5](#_Toc201834157)

[**2.**	**HPLC chromatogram**	S6](#_Toc201834158)

[**Figure S9**. RP-HPLC spectrum of peptide **1**	S6](#_Toc201834159)

[**3.**	**Mass spectrometry**	S7](#_Toc201834160)

[**Figure S10.** ESI-MS of peptide **1**	S7](#_Toc201834161)

[**4.**	**Thermogravimetric analysis**	S7](#_Toc201834162)

[**Figure S11**. Thermogravimetric analysis of peptide **1**	S7](#_Toc201834163)

[**5.**	**Theoretical calculations**	S8](#_Toc201834164)

[Table S1. Cartesian atom coordinates of peptide **1**	S8](#_Toc201834165)

# 1. **Spectroscopy characterization**

## 1.1. Spectra of peptide **1** (Mal-LARGDVGA)

## **Figure S1.** ^1^H NMR spectrum of peptide Mal-LARGDVGA.

## **Figure S2.** ^13^C NMR spectrum of peptide **1**.

**Figure S3.** DEPT spectrum of peptide Mal-LARGDVGA.

## **Figure S4.** HSQC spectrum of peptide **1**

## **Figure S5.** HMBC spectrum of peptide **1**

## **Figure S6.** COSY spectrum of peptide **1**.

## **Figure S7.** ^15^N-^1^H correlation HMBC spectrum of peptide **1**

## **Figure S8.** FTIR spectrum of peptide **1**.

# 2. **HPLC chromatogram**

**Figure S9**. RP-HPLC spectrum of peptide **1**, *t_R_* 11.0 min. (TFA:acetonitrile, 9.5:0.5, flow rate 0.8 mL/min).

# 3. **Mass spectrometry**

## **Figure S10.** ESI-MS of peptide **1**

# 4. **Thermogravimetric analysis**

## **Figure S11**. Thermogravimetric analysis of peptide **1**

# 5. **Theoretical calculations**

Table S1. Cartesian atom coordinates of peptide **2**.

| **Number** | **Atom** | **Residue** | **X** | **Y** | **Z** |
| --- | --- | --- | --- | --- | --- |
| 1 | C | LEU 1 | -2.257 | -0.334 | -3.230 |
| 2 | C |  | -4.016 | -0.080 | -4.975 |
| 3 | C |  | -3.748 | -0.091 | -3.473 |
| 4 | C |  | -4.637 | -1.115 | -2.773 |
| 5 | C |  | -1.876 | -0.429 | -1.758 |
| 6 | C |  | -0.407 | -0.718 | -1.431 |
| 7 | O |  | -0.137 | -1.141 | -0.313 |
| 8 | N |  | -2.332 | 0.696 | -0.960 |
| 9 | C | ALA 2 | 2.441 | -1.758 | -3.223 |
| 10 | C |  | 1.914 | -0.784 | -2.176 |
| 11 | C |  | 2.792 | 0.470 | -2.090 |
| 12 | N |  | 0.513 | -0.501 | -2.378 |
| 13 | O |  | 3.999 | 0.386 | -2.227 |
| 14 | C | ARG 3 | 2.581 | 3.612 | -0.413 |
| 15 | C |  | 2.859 | 2.902 | -1.741 |
| 16 | N |  | 2.167 | 1.630 | -1.778 |
| 17 | O |  | 2.075 | 4.716 | -0.363 |
| 18 | N |  | -0.344 | 2.907 | -4.624 |
| 19 | N |  | 0.549 | 0.828 | -4.984 |
| 20 | C |  | 0.566 | 2.093 | -5.206 |
| 21 | N |  | 1.450 | 2.744 | -6.066 |
| 22 | C |  | 2.330 | 3.763 | -5.494 |
| 23 | C |  | 3.104 | 3.315 | -4.242 |
| 24 | C |  | 2.479 | 3.788 | -2.926 |
| 25 | C | GLY 4 | 3.725 | 1.784 | 0.892 |
| 26 | C |  | 3.009 | 0.564 | 1.440 |
| 27 | O |  | 3.658 | -0.470 | 1.589 |
| 28 | N |  | 2.923 | 2.965 | 0.734 |
| 29 | O | ASP 5 | 2.932 | -2.795 | 2.711 |
| 30 | C |  | -0.480 | -0.129 | 2.394 |
| 31 | C |  | 1.007 | -0.459 | 2.275 |
| 32 | O |  | 1.024 | -3.350 | 3.667 |
| 33 | O |  | -0.936 | 0.934 | 2.037 |
| 34 | N |  | 1.730 | 0.689 | 1.796 |
| 35 | C |  | 1.833 | -2.500 | 3.367 |
| 36 | C |  | 1.587 | -1.027 | 3.602 |
| 37 | C | VAL 6 | -3.322 | -1.358 | 1.758 |
| 38 | C |  | -2.632 | -1.181 | 3.119 |
| 39 | C |  | -2.389 | 0.234 | 5.187 |
| 40 | O |  | -3.919 | -0.441 | 1.220 |
| 41 | N |  | -1.193 | -1.142 | 2.930 |
| 42 | C |  | -3.218 | -0.021 | 3.933 |
| 43 | C |  | -4.660 | -0.342 | 4.319 |
| 44 | C | GLY 7 | -2.415 | -3.697 | 1.479 |
| 45 | C |  | -1.458 | -3.921 | 0.310 |
| 46 | O |  | -1.845 | -3.761 | -0.831 |
| 47 | N |  | -3.280 | -2.583 | 1.191 |
| 48 | C | ALA 8 | 2.008 | -5.213 | 0.162 |
| 49 | C |  | 0.762 | -4.551 | -0.418 |
| 50 | C |  | 0.252 | -5.398 | -1.569 |
| 51 | N |  | -0.230 | -4.364 | 0.617 |
| 52 | O |  | 0.579 | -5.225 | -2.710 |
| 53 | O |  | -0.499 | -6.421 | -1.163 |
| 54 | C | Mal | -1.790 | 1.908 | -1.022 |
| 55 | C |  | -2.409 | 2.958 | -0.118 |
| 56 | O |  | -0.868 | 2.197 | -1.782 |
| 57 | C |  | -1.524 | 4.179 | -0.123 |
| 58 | O |  | -1.745 | 5.175 | -0.751 |
| 59 | O |  | -0.452 | 4.002 | 0.645 |
| 60 | H | LEU 1 | -1.690 | 0.439 | -3.749 |
| 61 | H |  | -1.970 | -1.280 | -3.702 |
| 62 | H |  | -3.385 | 0.648 | -5.486 |
| 63 | H |  | -3.813 | -1.058 | -5.417 |
| 64 | H |  | -5.056 | 0.169 | -5.190 |
| 65 | H |  | -4.001 | 0.900 | -3.083 |
| 66 | H |  | -5.680 | -0.989 | -3.067 |
| 67 | H |  | -4.606 | -1.020 | -1.686 |
| 68 | H |  | -4.343 | -2.135 | -3.031 |
| 69 | H |  | -2.390 | -1.298 | -1.348 |
| 70 | H |  | -2.997 | 0.496 | -0.223 |
| 71 | H | ALA 2 | 3.485 | -1.998 | -3.036 |
| 72 | H |  | 1.861 | -2.680 | -3.198 |
| 73 | H |  | 2.369 | -1.343 | -4.228 |
| 74 | H |  | 2.009 | -1.257 | -1.194 |
| 75 | H |  | 0.260 | -0.101 | -3.289 |
| 76 | H | ARG 3 | 3.931 | 2.683 | -1.795 |
| 77 | H |  | 1.153 | 1.661 | -1.781 |
| 78 | H |  | -0.351 | 3.885 | -4.843 |
| 79 | H |  | -0.751 | 2.633 | -3.743 |
| 80 | H |  | 1.321 | 0.364 | -5.445 |
| 81 | H |  | 1.981 | 2.075 | -6.603 |
| 82 | H |  | 3.021 | 4.039 | -6.291 |
| 83 | H |  | 1.759 | 4.671 | -5.284 |
| 84 | H |  | 3.195 | 2.226 | -4.263 |
| 85 | H |  | 4.129 | 3.691 | -4.293 |
| 86 | H |  | 2.793 | 4.814 | -2.732 |
| 87 | H |  | 1.391 | 3.820 | -2.992 |
| 88 | H | GLY 4 | 4.204 | 1.488 | -0.040 |
| 89 | H |  | 4.547 | 1.990 | 1.583 |
| 90 | H |  | 2.766 | 3.520 | 1.563 |
| 91 | H | ASP 5 | 3.341 | -1.975 | 2.336 |
| 92 | H |  | 1.052 | -1.226 | 1.497 |
| 93 | H |  | 1.192 | 1.505 | 1.528 |
| 94 | H |  | 0.898 | -0.895 | 4.435 |
| 95 | H |  | 2.505 | -0.508 | 3.873 |
| 96 | H | VAL 6 | -2.811 | -2.088 | 3.707 |
| 97 | H |  | -2.862 | 1.003 | 5.798 |
| 98 | H |  | -2.304 | -0.664 | 5.805 |
| 99 | H |  | -1.383 | 0.584 | 4.960 |
| 100 | H |  | -0.682 | -1.953 | 3.245 |
| 101 | H |  | -3.215 | 0.880 | 3.320 |
| 102 | H |  | -5.301 | -0.489 | 3.452 |
| 103 | H |  | -5.088 | 0.477 | 4.898 |
| 104 | H |  | -4.714 | -1.239 | 4.942 |
| 105 | H | GLY 7 | -1.891 | -3.561 | 2.421 |
| 106 | H |  | -3.012 | -4.607 | 1.591 |
| 107 | H |  | -3.704 | -2.624 | 0.274 |
| 108 | H | ALA 8 | 2.438 | -4.602 | 0.955 |
| 109 | H |  | 1.780 | -6.197 | 0.571 |
| 110 | H |  | 2.764 | -5.328 | -0.612 |
| 111 | H |  | 1.031 | -3.582 | -0.847 |
| 112 | H |  | 0.078 | -4.400 | 1.577 |
| 113 | H |  | -0.769 | -6.922 | -1.943 |
| 114 | H | Mal | -3.392 | 3.228 | -0.507 |
| 115 | H |  | -2.537 | 2.558 | 0.889 |
| 116 | H |  | 0.198 | 4.699 | 0.457 |

Table S2. Cartesian atom coordinates of peptide **2**.

| **Number** | **Atom** | **Residue** | **X** | **Y** | **Z** |
| --- | --- | --- | --- | --- | --- |
| 1 | H | ALA 1 | -5.611 | 7.052 | -1.650 |
| 2 | C |  | -7.613 | 6.699 | -0.130 |
| 3 | C |  | -6.307 | 5.912 | -0.037 |
| 4 | C |  | -6.555 | 4.469 | -0.495 |
| 5 | N |  | -5.299 | 6.559 | -0.827 |
| 6 | O |  | -6.369 | 4.114 | -1.635 |
| 7 | C | GLY 2 | -7.062 | 2.229 | 0.228 |
| 8 | C |  | -5.643 | 1.666 | 0.325 |
| 9 | N |  | -7.058 | 3.646 | 0.467 |
| 10 | O |  | -4.847 | 2.159 | 1.095 |
| 11 | C | VAL 3 | -4.190 | -0.177 | -0.485 |
| 12 | C |  | -4.216 | -1.161 | 0.689 |
| 13 | N |  | -5.390 | 0.626 | -0.491 |
| 14 | O |  | -5.039 | -1.119 | 1.577 |
| 15 | C |  | -1.973 | 0.102 | -1.703 |
| 16 | C |  | -2.078 | 0.782 | 0.709 |
| 17 | C |  | -2.869 | 0.648 | -0.592 |
| 18 | C | ASP 4 | -2.937 | -2.958 | 1.740 |
| 19 | C |  | -1.482 | -3.405 | 1.552 |
| 20 | N |  | -3.255 | -2.113 | 0.621 |
| 21 | O |  | -0.869 | -3.159 | 0.528 |
| 22 | O |  | -4.955 | -5.767 | 3.251 |
| 23 | O |  | -3.316 | -4.594 | 4.105 |
| 24 | C |  | -4.011 | -4.829 | 3.157 |
| 25 | C |  | -3.925 | -4.133 | 1.817 |
| 26 | C | GLY 5 | 0.453 | -4.408 | 2.547 |
| 27 | C |  | 1.319 | -3.156 | 2.691 |
| 28 | N |  | -0.942 | -4.095 | 2.568 |
| 29 | O |  | 0.898 | -2.160 | 3.225 |
| 30 | C | ARG 6 | 3.714 | -2.494 | 2.494 |
| 31 | C |  | 4.252 | -2.905 | 3.872 |
| 32 | N |  | 2.566 | -3.299 | 2.164 |
| 33 | O |  | 3.552 | -3.457 | 4.686 |
| 34 | C |  | 2.326 | 0.898 | 1.238 |
| 35 | C |  | 2.903 | -0.503 | 1.120 |
| 36 | N |  | 0.575 | 2.681 | -1.402 |
| 37 | N |  | 0.768 | 3.271 | 0.813 |
| 38 | C |  | 1.077 | 2.482 | -0.154 |
| 39 | N |  | 1.886 | 1.375 | -0.054 |
| 40 | C |  | 3.449 | -0.981 | 2.459 |
| 41 | C | ALA 7 | 7.608 | -1.295 | 4.002 |
| 42 | C |  | 6.585 | -2.142 | 3.249 |
| 43 | C |  | 7.317 | -3.27 | 2.517 |
| 44 | N |  | 5.553 | -2.589 | 4.153 |
| 45 | O |  | 8.065 | -2.986 | 1.588 |
| 46 | C |  | 7.747 | -5.729 | 2.449 |
| 47 | C | LEU 8 | 9.277 | -5.754 | 2.617 |
| 48 | N |  | 7.128 | -4.512 | 2.972 |
| 49 | O |  | 9.814 | -6.654 | 3.194 |
| 50 | C |  | 4.825 | -5.741 | 1.122 |
| 51 | C |  | 5.819 | -8.018 | 1.490 |
| 52 | C |  | 5.980 | -6.677 | 0.781 |
| 53 | C |  | 7.367 | -6.058 | 0.997 |
| 54 | C |  | -3.954 | 6.317 | -0.811 |
| 55 | O |  | 9.961 | -4.780 | 2.045 |
| 56 | C | Mal | -3.438 | 5.459 | 0.329 |
| 57 | O |  | -3.215 | 6.835 | -1.613 |
| 58 | C |  | -2.096 | 4.834 | 0.016 |
| 59 | O |  | -1.225 | 4.990 | 0.974 |
| 60 | O |  | -1.911 | 4.207 | -1.005 |
| 61 | H |  | 9.373 | -4.093 | 1.661 |
| 62 | H | ALA 1 | -7.980 | 6.718 | -1.157 |
| 63 | H |  | -8.387 | 6.247 | 0.488 |
| 64 | H |  | -5.992 | 5.910 | 1.012 |
| 65 | H |  | -7.524 | 2.029 | -0.739 |
| 66 | H |  | -0.404 | 4.396 | 0.838 |
| 67 | H | GLY 2 | -7.675 | 1.742 | 0.989 |
| 68 | H |  | -6.919 | 3.923 | 1.425 |
| 69 | H |  | -4.259 | -0.784 | -1.394 |
| 70 | H | VAL 3 | -6.142 | 0.307 | -1.075 |
| 71 | H |  | -1.116 | 0.762 | -1.836 |
| 72 | H |  | -1.579 | -0.895 | -1.481 |
| 73 | H |  | -2.498 | 0.049 | -2.659 |
| 74 | H |  | -1.389 | 1.620 | 0.620 |
| 75 | H |  | -2.719 | 0.989 | 1.562 |
| 76 | H |  | -1.482 | -0.107 | 0.928 |
| 77 | H |  | -3.165 | 1.651 | -0.900 |
| 78 | H |  | -3.009 | -2.382 | 2.669 |
| 79 | H | ASP 4 | -2.537 | -2.061 | -0.087 |
| 80 | H |  | -5.471 | -5.833 | 2.441 |
| 81 | H |  | -3.707 | -4.867 | 1.034 |
| 82 | H |  | -4.917 | -3.728 | 1.594 |
| 83 | H |  | 0.705 | -4.961 | 1.639 |
| 84 | H |  | 0.675 | -5.06 | 3.395 |
| 85 | H |  | -1.450 | -4.149 | 3.439 |
| 86 | H | GLY 5 | 4.462 | -2.719 | 1.727 |
| 87 | H |  | 2.780 | -4.199 | 1.767 |
| 88 | H |  | 1.499 | 0.857 | 1.958 |
| 89 | H | ARG 6 | 3.077 | 1.584 | 1.642 |
| 90 | H |  | 3.694 | -0.521 | 0.364 |
| 91 | H |  | 2.114 | -1.176 | 0.776 |
| 92 | H |  | 1.166 | 2.510 | -2.195 |
| 93 | H |  | -0.137 | 3.396 | -1.496 |
| 94 | H |  | 1.063 | 2.939 | 1.720 |
| 95 | H |  | 1.626 | 0.642 | -0.696 |
| 96 | H |  | 2.763 | -0.719 | 3.262 |
| 97 | H |  | 4.387 | -0.459 | 2.671 |
| 98 | H |  | 7.119 | -0.434 | 4.455 |
| 99 | H |  | 8.096 | -1.869 | 4.792 |
| 100 | H | ALA 7 | 8.380 | -0.937 | 3.325 |
| 101 | H |  | 6.153 | -1.516 | 2.467 |
| 102 | H |  | 5.812 | -2.723 | 5.121 |
| 103 | H |  | 7.393 | -6.530 | 3.095 |
| 104 | H |  | 6.511 | -4.620 | 3.760 |
| 105 | H | LEU 8 | 3.881 | -6.174 | 0.786 |
| 106 | H |  | 4.74 | -5.572 | 2.197 |
| 107 | H |  | 4.942 | -4.773 | 0.629 |
| 108 | H |  | 6.639 | -8.698 | 1.254 |
| 109 | H |  | 5.784 | -7.910 | 2.576 |
| 110 | H |  | 4.891 | -8.505 | 1.192 |
| 111 | H |  | 5.923 | -6.871 | -0.295 |
| 112 | H |  | 8.102 | -6.779 | 0.626 |
| 113 | H |  | -7.462 | 7.725 | 0.203 |
| 114 | H | Mal | 7.476 | -5.172 | 0.369 |
| 115 | H |  | -3.372 | 6.077 | 1.228 |
| 116 | H |  | -4.106 | 4.628 | 0.570 |
